# Work Engagement Among Advanced Practice Nurses (APNs) in Japan: The Role of Supervisory and Organizational Support

**DOI:** 10.1111/inr.70130

**Published:** 2025-11-20

**Authors:** Kazumi Kubota, Kei Sato, Yuri Suenaga

**Affiliations:** ^1^ Research Orgnization Shimonoseki City University Yamaguchi Japan; ^2^ Department of Healthcare Information Management The University of Tokyo Hospital Tokyo Japan; ^3^ Health and Global Policy Institute Tokyo Japan; ^4^ Department of Biostatistics School of Medicine Yokohama City University Yokohama Japan; ^5^ Department of Nursing Showa Medical University Hospital Tokyo Japan; ^6^ Division of Nursing Management Postgraduate School of Healthcare Tokyo Healthcare University Tokyo Japan

**Keywords:** advanced practice nurses (APNs), certified nurses (CNs), nursing management, organizational factors, supervisor support, work engagement

## Abstract

**Aim:**

To examine how personal, social support, and organizational factors influence work engagement (WE) among advanced practice nurses (APNs; certified nurses, CNs) in Japan and whether supervisory structure (unified vs. non‐unified) moderates these relationships.

**Background:**

APNs (CNs) play a vital role in specialized healthcare, but factors influencing their WE remain underexplored. Previous studies suggest that supervisor support and organizational factors impact nurses’ professional motivation and retention.

**Methods:**

A cross‐sectional web‐based survey was conducted among 307 APNs (CNs) from six nursing specialties in Japan. WE was assessed using the Ultra‐Short Utrecht Work Engagement Scale (UWES‐3). Independent variables included supervisor support (emotional, evaluative, informational, procedural), organizational factors (trust with management, fair evaluation, respect for individuals, career development). Hierarchical multiple regression analyses were performed separately for the unified and non‐unified supervisory groups.

**Results:**

Evaluative support positively influenced WE across both groups, emphasizing the importance of structured feedback and professional recognition. Emotional support was negatively associated with WE in the unified group, suggesting that excessive reliance on supervisors may reduce autonomy. Organizational factors did not significantly impact WE, and supervisory structure did not moderate these relationships.

**Conclusions:**

While APNs (CNs) benefit from structured supervisor support, broader organizational policies may be less influential. Prioritizing performance evaluation systems and mentorship programs could enhance APNs’ (CNs’) engagement and retention.

**Implications for Nursing and Health Policy:**

Healthcare institutions should implement targeted leadership training and structured evaluation systems to sustain APNs’ (CNs’) motivation and professional development.

## 1 Introduction

Advanced practice nurses (APNs), known in Japan as certified nurses (CNs), specialize in advanced clinical fields to enhance healthcare quality and patient outcomes. While globally the term APN encompasses roles such as nurse practitioners and clinical nurse specialists, Japanese CNs are most akin to clinical nurse specialists (CNSs). Their role primarily emphasizes advanced clinical expertise within hospital settings, focusing on direct patient care, interdisciplinary collaboration, and clinical education (Japan Nursing Association 2025), rather than expanded practice authority. Unlike Nurse Practitioners in many Western countries, who can diagnose conditions and prescribe medications independently, Japanese APNs (CNs) focus on advanced nursing expertise within the traditional scope of nursing practice. Japanese CNs fulfill the ICN definition of APNs through their specialized clinical practice activities such as managing complex patient care trajectories, providing expert consultation across departments, developing evidence‐based protocols within their specialty areas, and leading educational initiatives for nursing staff. This aligns with the International Council of Nurses' (ICN) definition of APNs, which includes roles that involve the application of advanced knowledge and skills in clinical practice, education, research, and management to improve patient outcomes (ICN 2020). As of 2023, there were 24,095 registered CNs, marking a 14% increase from 2021, reflecting growing demand for specialized care (Japan Nursing Association 2025).

Work engagement (WE), characterized by vigor, dedication, and absorption (Schaufeli et al. 2019), is a critical psychological state for healthcare professionals, significantly impacting job satisfaction, retention, and quality of patient care (Naef et al. 2021). In Japan, fostering high WE among nurses is essential for a sustainable healthcare system, particularly given the increasing demands and complexities of clinical practice. Recent studies indicate that Japanese nurses face unique challenges affecting their engagement, including high workloads, hierarchical organizational structures, and limited career advancement opportunities (Fukazaki and Iwata 2024; Kuribayashi et al. 2025). Within this context, understanding factors that promote WE is crucial for addressing nursing shortages and improving healthcare outcomes.

APNs (CNs) function within complex organizational structures, often reporting to multiple supervisors responsible for different aspects of their clinical and leadership duties (Caroccini et al. 2024). While some APNs (CNs) operate under a unified supervisor who oversees both their general nursing duties and APNs (CNs)‐specific responsibilities, others report to non‐unified supervisory systems, where separate supervisors manage different aspects of their role (Wanning et al. 2024). This fragmentation may contribute to role ambiguity, inconsistent feedback, and increased job strain, which can subsequently impact WE (Kuribayashi et al. 2025). In addition to supervisor support, organizational factors such as workplace culture, career development pathways, and institutional trust have been examined as potential contributors to WE in healthcare professionals (Fukazaki and Iwata 2024). However, studies suggest that APNs (CNs) may place greater emphasis on immediate supervisory feedback than on broader institutional support systems (Yang et al. 2023). These findings indicate that personal and supervisory factors may have a stronger impact on WE compared with institutional‐level support mechanisms.

While previous studies have explored the role of supervisory and organizational support in nursing WE, most have focused on Western healthcare systems, particularly in the United States and Europe, where the role of APNs is well‐established (Fajarini et al. 2025; Slade et al. 2024). In contrast, Japan's CN system is relatively unique, as it does not grant expanded practice authority but instead emphasizes advanced clinical expertise within hospital settings (Japan Nursing Association 2025).

Given the increasing importance of APNs (CNs) in Japan's healthcare system, this study aims to examine how personal factors, supervisor support, and organizational factors influence WE among APNs (CNs). Additionally, we explore whether our findings align with international research on nurse engagement or reveal unique trends specific to Japan's hierarchical healthcare structure.

Additionally, it investigates whether the type of supervisory structure—unified vs. non‐unified—moderates the relationship between support systems and WE. Understanding these dynamics will provide critical insights into optimizing APNs’ (CNs’) work environments to promote engagement, professional fulfillment, and retention.

## 2 Methods

### 2.1 Participants

This study employed a cross‐sectional, web‐based survey targeting certified APNs (CNs) in Japan. A stratified sampling method was used to select 1,000 APNs (CNs) affiliated with hospitals across Japan. The stratification was based on six nursing specialties with over 1,000 registered APNs (CNs) in Japan (i.e., cancer chemotherapy nursing, palliative care, emergency nursing, intensive care, dementia nursing, and wound, ostomy, and continence nursing). Infection control APNs (CNs) were excluded due to the unique impact of the COVID‐19 pandemic on their roles and WE, which may have differed substantially from other specialties (Japan Nursing Association 2025; Tsuchihashi et al. 2024). The inclusion criteria for participants were: (1) being an active APNs (CNs) in one of the six selected specialties, (2) working in a hospital setting, and (3) having at least one supervisor in their clinical practice. We excluded APNs (CNs) affiliated with the researchers’ institution to prevent conflicts of interest.

### 2.2 Data Collection

Data were collected from July 1 to August 31, 2020. The survey invitation was distributed via postal mail to the selected APNs (CNs). The letter included a description of the study and a URL link to the online questionnaire. Participants accessed the survey anonymously and voluntarily. A total of 334 responses were received. After excluding cases with missing data, 307 responses were included in the final analysis (valid response rate: 30.7%).

### 2.3 Measures

The questionnaire consisted of five major domains: demographic variable, supervisor characteristics, supervisor support, organizational factors, and work engagement. The details of these domains are as follows:

#### 2.3.1 Demographic Variables

We collected background characteristics including age, years of clinical nursing experience, years of experience as APNs (CNs), and job position (management vs. non‐management).

#### 2.3.2 Supervisor Characteristics

APNs (CNs) engage in a wide range of professional activities, including bedside nursing care as ward staff, specialized APNs (CNs) activities, interdisciplinary team collaboration, and consultation services across different hospital departments. Due to the diverse nature of APNs (CNs) roles, APNs (CNs) often have multiple supervisors overseeing different aspects of their work. In this study, we defined two types of supervisors: (1) APNs (CNs) activity supervisor: A supervisor responsible for the APNs (CNs) specialized activities. (2) Non‐APNs (CNs) activity supervisor: A supervisor overseeing general nursing duties within the hospital. Based on this classification, supervisors’ perspectives were categorized as follows: (1) Unified: The APNs (CNs) has a single, consistent supervisor overseeing both general clinical practice and APNs (CNs)‐specific activities, and (2) Not Unified: The APNs (CNs) has multiple supervisors, with one responsible for general nursing duties and another overseeing APNs (CNs)‐specific activities.

#### 2.3.3 Supervisor Support

Supervisor support was assessed using the Nurse Workplace Support Scale, developed by Ida and Fukuda (2004). This scale was originally designed to measure both supervisor and coworker support, but the present study focused solely on supervisor support. The scale consists of four subscales: (1) emotional support (e.g., “My supervisor listens to my concerns and empathizes with my difficulties.”), (2) evaluative support (e.g., “My supervisor acknowledges and values my contributions.”), (3) informational support (e.g., “My supervisor provides specific and appropriate guidance.”), and (4) procedural (instrumental) support (e.g., “When I encounter a task I cannot complete alone, my supervisor willingly helps me.”). Each item was rated on a 5‐point Likert scale (1 = never, 5 = always). Previous studies have confirmed its construct validity, with path coefficients ranging from 0.82 to 0.99 (Ida and Fukuoka. 2004). Although internal reliability was not originally reported, the scale's validity is well‐established. Cronbach's α coefficients were 0.92, 0.96, 0.89, and 0.94 for emotional support, evaluative support, informational support, and procedural (instrumental) support, respectively.

#### 2.3.4 Organizational Factor

To evaluate organizational support beyond APNs (CNs)‐specific duties, we utilized four items from the Brief Job Stress Questionnaire (BJSQ), developed by Inoue et al. (2014)​, as follows: Trust with management (e.g., “The management team takes employees’ suggestions seriously.”), Fair personal evaluation (e.g., “My colleagues treat me fairly in the workplace.”), Respect for Individual (e.g., “My workplace respects individual values.”), and career development (e.g., “My workplace offers training that enhances motivation and career development.”). This scale assesses psychosocial workplace factors at both the departmental and institutional levels, focusing on workplace resources that promote engagement and well‐being. Responses were recorded on a 4‐point Likert scale (1 = strongly disagree, 4 = strongly agree). Content validity was confirmed through expert review and pretesting with APNs (CNs). For the four items used from the BJSQ, internal consistency in this study was acceptable, with Cronbach's α values of 0.78 for trust with management, 0.75 for fair personal evaluation, 0.79 for respect for individuals, and 0.76 for career development.

#### 2.3.5 Work Engagement

WE was assessed using the Ultra‐Short Utrecht Work Engagement Scale (UWES‐3), developed by Schaufeli et al. (2019). While the original UWES (Schaufeli et al. 2008) and its Japanese validation (Shimazu et al. 2008) have been widely used, the UWES‐3 was selected in this study due to its validated brevity and cross‐cultural applicability. The UWES‐3 consists of three core dimensions: (1) vigor (e.g., “At my work, I feel bursting with energy.”), (2) Dedication (e.g., “I am enthusiastic about my job.”), and absorption (e.g., “I am immersed in my work.”). Each item was rated on a 7‐point Likert scale (0 = never, 6 = always). The UWES‐3 has been validated across five countries, including Japan, demonstrating strong internal consistency (Cronbach's α = 0.91 in our sample) and construct validity   (Schaufeli et al. 2019).

### 2.4 Statistical Analysis

To describe the background characteristics of the participants, we calculated descriptive statistics for demographic variables, supervisor characteristics, supervisor support, organizational factors, and WE. Given that the level of support received may vary depending on supervisor attributes, we categorized respondents based on their supervisor's characteristics and restricted our sample to those in a position equivalent to or below the chief nurse level. The final analysis included 307 respondents who reported having a supervisor for APN (CN) activities.

A hierarchical multiple regression analysis was conducted using SPSS version 25, with WE as the dependent variable. To examine differences based on supervisor characteristics, we conducted the analysis separately for two supervisor groups: “Unified” and “Not Unified.” Predictor variables were entered in three steps. In Step 1, the model included years of clinical nursing experience, years of clinical experience as APNs (CNs), and job position categorized as management or non‐management. Step 2 included all variables from Step 1 with the addition of supervisor support, which consisted of emotional support, evaluative support, informational support, and procedural support. Step 3 included all variables from Step 2 along with organizational factors such as trust with management, fair personal evaluation, respect for individuals, and career development. By comparing the regression models between the Unified and Not Unified supervisor groups, we assessed whether the factors influencing WE differed based on supervisor characteristics. To assess multicollinearity among independent variables, the variance inflation factor (VIF) was examined. The results confirmed that VIF values did not exceed 10, indicating that multicollinearity was not a concern in this. A formal a priori power analysis was not conducted before data collection. However, a post‐hoc power analysis using G*Power 3.1 for multiple regression with 11 predictors (our most complex model), α = 0.05, and a medium effect size (*f* ^2^ = 0.15) indicated that our sample sizes (177 in the unified group and 130 in the non‐unified group) provided adequate power (>0.80) for detecting meaningful effects.

### 2.5 Ethical Considerations

This study was approved by the Human Research Ethics Committee of Tokyo Healthcare University (approval numbers: In‐32‐15B and In‐32‐36) and conducted in accordance with the ethical guidelines for research involving human subjects. Participants were informed that participation was voluntary, and declining to participate would not result in any disadvantages. The questionnaire was anonymous, and since no identifiable personal information was collected, withdrawal of consent after submission was not possible. Collected data were securely managed and used solely for research purposes. Participants provided informed consent by checking the agreement box before starting the survey.

## 3 Results

Table 1 presents the demographic characteristics of the 307 APNs (CNs) included in the study. The majority of participants were aged 40–49 years (51.8%), followed by those aged 30–39 years (24.8%) and 50 years or older (22.8%). On average, participants had 21.6 years (SD = 6.7) of clinical nursing experience, with 7.7 years (SD = 4.5) of experience as APNs (CNs). Regarding their area of specialization, wound, ostomy, and continence nursing was the most common field (26.1%), followed by palliative care (20.5%), cancer chemotherapy nursing (16.3%), dementia nursing (14.7%), emergency nursing (12.4%), and intensive care (10.1%). In terms of job position, 63.5% of participants held management roles, while 36.5% were in non‐management positions. Participants were also classified based on their supervisor's perspective, with 57.7% categorized as having a unified supervisor, meaning they had a single, designated supervisor in clinical practice, while 42.3% were in the not unified group, where multiple supervisors oversaw their clinical activities. The overall mean score for WE was 3.4 (SD = 1.1), suggesting a moderate level of engagement among participants. Regarding perceived support, the mean scores for supervisor support subscales were 8.0 (SD = 3.6) for emotional support, 12.4 (SD = 4.6) for evaluative support, 7.5 (SD = 3.3) for informational support, and 9.0 (SD = 4.5) for procedural support. In terms of organizational support, participants reported moderate levels across all dimensions, with mean scores of 2.9 (SD = 0.8) for trust in management, 2.8 (SD = 0.8) for fair personal evaluation, 2.9 (SD = 0.8) for respect for individuals, and 2.6 (SD = 0.8) for career development.

**TABLE 1:** Demographic characteristics among Japanese certified nurses (*N* = 307).

	*n*	(%)	Mean	(SD)
Age group				
20 or less	2	(0.7)		
30–39	78	(24.8)		
40–49	159	(51.8)		
50 or more	70	(22.8)		
Years of clinical nursing experience			21.6	(6.7)
Years of clinical experience as CNs^a)^			7.7	(4.5)
Specialized field as a CNs^a^				
Cancer chemotherapy nursing	50	(16.3)		
Palliative care	63	(20.5)		
Emergency nursing	38	(12.4)		
Intensive care	31	(10.1)		
Dementia nursing	45	(14.7)		
Wound, ostomy, and continence nursing	80	(26.1)		
Job position				
Management	195	(63.5)		
Non‐management	112	(36.5)		
Supervisor's perspective^b^				
Unified	177	(57.7)		
Not unified	130	(42.3)		
Supervisor support^c^				
Emotional support			8.0	(3.6)
Evaluative support			12.4	(4.6)
Informational support			7.5	(3.3)
Procedural support			9.0	(4.5)
Organizational support				
Trust with management			2.9	(0.8)
Fair personal evaluation			2.8	(0.8)
Respect for individuals			2.9	(0.8)
Career development			2.6	(0.8)
Work engagement (average)			3.4	(1.1)

^a^ CNs: Certified nurses.

^b^ Unified: The supervisor is unified in clinical practice. Not unified: There are multiple supervisors in clinical practice.

^c^ Calculate the total score for each subscale

Table 2 presents the results of hierarchical multiple regression analyses examining factors associated with WE among APNs (CNs) in the unified supervisor group. In Step 1, job position (management/non‐management) was significantly associated with WE, with APNs (CNs) in non‐management positions reporting higher engagement than those in management roles (β = −0.162, *p* = 0.043).

**TABLE 2:** Hierarchical multiple regression analyses predicting work engagement among Japanese CNs (unified group) (*N* = 177).

		*b*	SE	β	*t*	*p* value	95% CI	
Step 1	Years of clinical nursing experience	0.002	0.015	0.010	0.104	0.918	−0.028	0.031
	Years of clinical experience as CNs	0.020	0.023	0.079	0.870	0.386	−0.025	0.065
	Job position (management/non‐management)	−0.383	0.188	−0.162	−2.035	0.043	−0.755	−0.012
	Adjusted *R* ^2^: 0.042							
Step 2	Years of clinical nursing experience	0.009	0.014	0.058	0.646	0.519	−0.019	0.038
	Years of clinical experience as CNs	0.006	0.022	0.026	0.286	0.775	−0.038	0.050
	Job position (management/non‐management)	−0.359	0.183	−0.152	−1.959	0.052	−0.721	0.003
	Emotional support	−0.176	0.056	−0.582	−3.172	0.002	−0.286	−0.067
	Evaluative support	0.121	0.028	0.511	4.397	0.000	0.067	0.175
	Informational support	0.068	0.053	0.202	1.266	0.207	−0.038	0.173
	Procedural support	0.027	0.035	0.108	0.789	0.431	−0.041	0.096
	Adjusted *R* ^2^: 0.128							
Step 3	Years of clinical nursing experience	0.011	0.015	0.066	0.722	0.471	−0.018	0.039
	Years of clinical experience as CNs	0.001	0.023	0.003	0.035	0.972	−0.045	0.046
	Job position (management/non‐management)	−0.348	0.187	−0.147	−1.863	0.064	−0.717	0.021
	Emotional support	−0.172	0.056	−0.567	−3.060	0.003	−0.282	−0.061
	Evaluative support	0.112	0.029	0.471	3.870	0.000	0.055	0.169
	Informational support	0.048	0.057	0.142	0.833	0.406	−0.065	0.161
	Procedural support	0.035	0.036	0.139	0.975	0.331	−0.036	0.107
	Trust with management	−0.082	0.149	−0.056	−0.551	0.582	−0.376	0.212
	Fair personal evaluation	−0.111	0.165	−0.078	−0.670	0.504	−0.437	0.216
	Respect for individuals	0.049	0.163	0.033	0.304	0.761	−0.271	0.370
	Career development	−0.064	0.130	−0.045	−0.495	0.621	−0.321	0.192
	Adjusted *R* ^2^: 0.120							

Abbreviations: β, Standardized regression coefficients; *b*, Unstandardized regression coefficients; CI, Confidence interval; CNs, Certified nurses.

In Step 2, after adding supervisor support variables, the effect of job position became non‐significant (*p* = 0.052), suggesting that supervisor support may mediate this relationship. Emotional support showed a significant negative association with WE (β = −0.582, *p* = 0.002), whereas evaluative support was positively associated (β = 0.511, *p* < 0.001), indicating that structured feedback enhances engagement while emotional dependence on supervisors may lower it. Informational and procedural support were not significant predictors.　Step 3 introduced organizational support factors, but none of these variables were significantly associated with WE. The significant associations of emotional support (*p* = 0.003) and evaluative support (*p* < 0.001) remained unchanged, while job position remained non‐significant (*p* = 0.064).

Table 3 presents the results of hierarchical multiple regression analyses examining factors associated with WE among APNs (CNs) in the non‐unified supervisor group. In Step 1, job position (management/non‐management) was not significantly associated with WE (β = −0.092, *p* = 0.310), and neither years of clinical nursing experience nor years of clinical experience as APNs (CNs) showed significant effects. In Step 2, after adding supervisor support variables, none of the predictors were significantly associated with WE. Emotional support (β = 0.086, *p* = 0.695), evaluative support (β = 0.196, *p* = 0.203), informational support (β = 0.247, *p* = 0.170), and procedural support (β = −0.105, *p* = 0.525) did not reach statistical significance. Job position remained non‐significant (*p* = 0.635). Step 3 introduced organizational support factors, but none of these variables were significantly associated with WE. Emotional support (*p* = 0.599) and evaluative support (*p* = 0.353) remained non‐significant, while trust in management (*p* = 0.393), fair personal evaluation (*p* = 0.375), and career development (*p* = 0.793) also did not show significant effects.

**TABLE 3:** Hierarchical multiple regression analyses predicting work engagement among Japanese CNs (non‐unified group) (*N* = 130).

		*b*	SE	β	*t*	*p* value	95% CI	
Step 1	Years of clinical nursing experience	0.017	0.017	0.099	1.015	0.312	−0.016	0.051
	Years of clinical experience as CNs	−0.006	0.024	−0.024	−0.244	0.808	−0.054	0.042
	Job position (management/non‐management)	−0.206	0.202	−0.092	−1.020	0.310	−0.605	0.193
	Adjusted *R* ^2^: −0.004							
Step 2	Years of clinical nursing experience	0.012	0.016	0.071	0.770	0.443	−0.019	0.044
	Years of clinical experience as CNs	0.000	0.023	−0.001	−0.007	0.994	−0.045	0.045
	Job position (management/non‐management)	−0.091	0.192	−0.041	−0.476	0.635	−0.470	0.288
	Emotional support	0.026	0.067	0.086	0.393	0.695	−0.107	0.159
	Evaluative support	0.048	0.037	0.196	1.279	0.203	−0.026	0.122
	Informational support	0.084	0.061	0.247	1.381	0.170	−0.036	0.203
	Procedural support	−0.025	0.039	−0.105	−0.638	0.525	−0.101	0.052
	Adjusted *R* ^2^: 0.135							
Step 3	Years of clinical nursing experience	0.014	0.016	0.079	0.836	0.405	−0.019	0.046
	Years of clinical experience as CNs	−0.001	0.023	−0.003	−0.032	0.974	−0.047	0.045
	Job position (management/non‐management)	−0.108	0.199	−0.048	−0.541	0.589	−0.503	0.287
	Emotional support	0.036	0.069	0.119	0.527	0.599	−0.100	0.173
	Evaluative support	0.036	0.039	0.148	0.931	0.353	−0.041	0.113
	Informational support	0.077	0.062	0.226	1.234	0.220	−0.046	0.200
	Procedural support	−0.022	0.039	−0.093	−0.550	0.584	−0.100	0.056
	Trust with management	0.131	0.153	0.092	0.857	0.393	−0.171	0.433
	Fair personal evaluation	−0.154	0.173	−0.109	−0.890	0.375	−0.496	0.189
	Respect for individuals	−0.078	0.172	−0.055	−0.454	0.651	−0.419	0.263
	Career development	0.040	0.152	0.029	0.263	0.793	−0.262	0.342
	Adjusted *R* ^2^: 0.118							

Abbreviations: β, Standardized regression coefficients; *b*, Unstandardized regression coefficients; CI, Confidence interval; CNs, Certified nurses.

## 4 Discussion

This study highlights the crucial role of supervisor support in fostering WE among APNs (CNs) in Japan. The findings align with previous literature, demonstrating that evaluative support—structured feedback and professional recognition—significantly enhances WE (Tsuchihashi et al. 2024). These results are consistent with international evidence showing that relational/transformational leadership and structured, evaluative feedback are robust drivers of nurse WE across diverse health systems (Keyko et al. 2016; García‐Sierra et al. 2016; Cummings et al. 2018). This reinforces broader evidence indicating that high‐quality supervision strengthens professional motivation, job performance, and long‐term career satisfaction (Kuribayashi et al. 2025).

### 4.1 The Role of Supervisor Support in WE

Among the forms of supervisor support examined, evaluative support showed a strong positive association with WE, suggesting that APNs (CNs) thrive in environments where their contributions are recognized, and structured feedback is consistently provided (Sato et al. 2023). From a Job Demands–Resources perspective, high‐quality supervisory feedback functions as a proximal job resource with a direct motivational pathway to engagement (Bakker and Demerouti 2017; Lesener et al. 2019). Supervisors who acknowledge APN (CN) expertise, provide guidance on career progression, and integrate APNs (CNs) into hospital decision‐making processes contribute significantly to sustained engagement (Naef et al. 2021).

Interestingly, emotional support was negatively associated with WE in the unified supervisor group. While emotional support generally helps reduce workplace stress, excessive reliance on supervisors for reassurance may inadvertently limit APNs’ (CNs’) sense of autonomy and self‐efficacy (Shimazu et al. 2008). This finding suggests that emotional support should be provided in a way that fosters independent decision‐making rather than creating dependency. For instance, leadership training programs could encourage supervisors to balance emotional encouragement with opportunities for APNs (CNs) to exercise professional judgment. Future research should examine how different types of emotional support influence nurse engagement across various professional levels. While emotional support is generally beneficial for mitigating workplace stress, excessive reliance on it may lead APNs (CNs) to perceive their role as constrained rather than empowered (Fukazaki and Iwata 2024). This finding diverges from studies on general nursing populations, where emotional support has been positively correlated with higher job satisfaction and retention (Wanning et al. 2024). The discrepancy likely reflects the advanced practice nature of APNs (CNs), who prioritize professional recognition and role clarity over emotional reassurance (Caroccini et al. 2024). By contrast, reviews in general nursing samples frequently report positive associations between emotional support and work attitudes, suggesting that our pattern may reflect role‐specific dynamics among APNs (Simpson 2009; Bargagliotti 2012; Keyko et al. 2016).

### 4.2 Supervisor Structure: Unified vs. Non‐Unified Models

We initially hypothesized that APNs (CNs) under a unified supervisory structure would report higher WE due to clearer role expectations and consistent feedback. However, our results showed no significant difference in WE between the unified and non‐unified groups. This may suggest that APNs (CNs) develop adaptive strategies to manage multiple supervisory relationships, mitigating potential role ambiguity (Caroccini et al. 2024 ).

Additionally, APNs (CNs) working under non‐unified structures may compensate for fragmented supervision by seeking peer support or engaging in self‐directed professional development. Similar findings have been observed in other hierarchical professions, where informal networks often supplement formal leadership structures (Wanning et al. 2024). Future qualitative research could explore how APNs (CNs) navigate complex supervisory relationships and whether institutional policies can better support APNs (CNs) in non‐unified settings.

This finding suggests that the quality of supervisory interactions, rather than the structural organization of supervision, is the primary determinant of engagement (Tsuchihashi et al. 2024). This emphasis on relational leadership quality is echoed internationally, where supportive and transformational leadership styles consistently predict higher engagement and better workforce outcomes (Cummings et al. 2018; Keyko et al. 2016). Previous literature has suggested that fragmented supervision could contribute to role ambiguity and reduce engagement, but the present study indicates that APNs (CNs) in non‐unified structures may develop adaptive strategies to manage multiple supervisory relationships, mitigating potential job strain (Kuribayashi et al. 2025).

### 4.3 Organizational Factors and WE

Contrary to hypotheses, organizational factors such as trust in management, fair evaluation, and career development did not show significant associations with WE. One possible explanation is that APNs (CNs) prioritize direct supervisory relationships over broader institutional policies due to the hierarchical nature of Japanese healthcare institutions (Kuribayashi et al. 2025). In contrast, previous studies in the United States and Europe have found stronger associations between organizational support and WE, likely reflecting differences in workplace autonomy and decision‐making structures (Yang et al. 2023). Additionally, our study sample included a high proportion of APNs (CNs) in management positions (63.5%), who may perceive organizational policies differently from non‐management nurses. Future studies should investigate whether perceptions of organizational support vary by role seniority and institutional type. Cross‐national evidence from the RN4CAST program documents substantial variation in nurse practice environments, staffing, and governance across European countries, with corresponding differences in workforce outcomes (Aiken et al. 2013; Heinen et al. 2013). Such contextual variability may help explain why, in our Japanese sample, proximal supervisory relationships appear more salient for WE than broader institutional policies.

This suggests that APNs (CNs) prioritize direct supervisory relationships over broader institutional policies when it comes to their engagement levels (Yang et al. 2023). Workplace culture variations and differences in hospital policies may partially explain these findings (Satoh et al. 2024). While prior research has indicated that organizational‐level support plays a role in reducing nurse burnout, our results imply that APNs (CNs), as specialized professionals, may rely more on immediate feedback mechanisms than on generalized institutional frameworks (Kuribayashi et al. 2025). Nevertheless, evidence indicates that perceived organizational support is generally beneficial for employee attitudes and well‐being; therefore, our null organizational effects should be interpreted with caution (Rhoades and Eisenberger 2002). Additionally, data were collected during the COVID‐19 pandemic (July–August 2020). Although infection control CNs were excluded, the extraordinary context may have influenced both WE and perceptions of supervisory and organizational support (e.g., workload fluctuations, changes in role expectations, and altered supervisory practices). Therefore, the present associations should be interpreted with caution, and future studies conducted in post‐pandemic settings are warranted to examine the stability of these findings.

## 5 Implications for Nursing and Health Policy

These findings have several implications for hospital administrators, nursing managers, and policymakers. Given the strong association between evaluative support and WE, institutions should implement structured mentorship programs, career development initiatives, and transparent performance evaluations to enhance APNs’ (CNs’) professional fulfillment (Fajarini et al. 2025). Additionally, leadership training for APN (CN) supervisors should emphasize the importance of constructive feedback and professional recognition, ensuring that APNs (CNs) receive consistent support that reinforces autonomy and skill development (Slade et al. 2024). While emotional support remains valuable, it should be offered in ways that empower APNs (CNs) rather than creating dependency (Sato et al. 2023). These recommendations align with international evidence that supportive/transformational leadership, clear performance feedback, and mentorship structures are associated with higher nurse engagement and improved workforce outcomes (Cummings et al. 2018; Keyko et al. 2016).

## 6 Limitations and Future Research

This study has several limitations. First, the cross‐sectional design precludes causal inferences, highlighting the need for longitudinal research to examine how supervisory and organizational factors impact WE over time (Fukazaki and Iwata 2024). Second, sampling and generalizability warrant caution: although we used stratified sampling across six APN (CN) specialties, we did not conduct a formal a priori power analysis. Future research should employ prospective power calculations and broaden the specialty coverage to enhance representativeness. Third, the timing of data collection (July–August 2020) coincided with the COVID‐19 pandemic. Even though infection control APNs (CNs) were excluded, pandemic‐related organizational changes and stressors may have affected both WE and perceptions of supervisory/organizational support and may also have contributed to the relatively low response rate (30.7%). Replication under post‐pandemic conditions is warranted. Fourth, differences in institutional policies may have influenced self‐reported supervisor support and WE levels, necessitating comparative studies across diverse hospital settings (Yang et al. 2023).

## 7 Conclusion

Overall, this study contributes to the growing body of literature on WE among APNs (CNs), emphasizing the critical role of structured supervisor support in sustaining engagement, motivation, and professional satisfaction. By prioritizing high‐quality evaluative support and career development opportunities, healthcare institutions can enhance APNs’ (CNs’) professional fulfillment, retention, and leadership potential, ultimately improving patient care outcomes in Japan's evolving healthcare landscape.

## Author Contributions

Study design: KK, KS, and YS. Data collection: KS. Data analysis: KK. Manuscript writing (draft): KK and KS. Manuscript writing (revised): KK and KS. Study supervision: YS. Critical revisions for important intellectual content: YS.

## Funding

This study was funded by the Tokyo Healthcare University Research Grant. The funding body had no role in the study design, data collection, analysis, interpretation, or manuscript preparation.

## Conflicts of Interest

The authors declare no conflicts of interest.

## Permission for Use of Scales

Permissions for the use of the Nurse Workplace Support Scale (Ida and Fukuda 2004) and selected items from the BJSQ (Inoue et al. 2014) were obtained from the respective original authors.

## Data Availability

The data that support the findings of this study are available from the corresponding author upon reasonable request.

## References

[inr70130-bib-0001] Aiken, L. H. , D. M. Sloane , L. Bruyneel , K. Van den Heede , and W. Sermeus ; RN4CAST Consortium . 2013. “Nurses' Reports of Working Conditions and Workforce Outcomes in 12 European Countries.” International Journal of Nursing Studies 50, no. 2: 143–153. 10.1016/j.ijnurstu.2012.11.009. 23254247

[inr70130-bib-0002] Bakker, A. B. , and E. Demerouti . 2017. “Job Demands–Resources Theory: Taking Stock and Looking Forward.” Journal of Occupational Health Psychology 22, no. 3: 273–285. 10.1037/ocp0000056. 27732008

[inr70130-bib-0003] Bargagliotti, L. A. 2012. “Work Engagement in Nursing: A Concept Analysis.” Journal of Advanced Nursing 68, no. 6: 1414–1428. 10.1111/j.1365-2648.2012.05912.x. 22044047

[inr70130-bib-0004] Caroccini, T. P. , A. P. Balsanelli , and V. Neves . 2024. “The Meaning of Work for Hospital Unit Nurses: A Scoping Review.” Revista Brasileira de Medicina do Trabalho 22, no. 3: e20231116. 10.47626/1679-4435-2023-1116. 39606771 PMC11595381

[inr70130-bib-0005] Cummings, G. G. , K. Tate , S. Lee , et al. 2018. “Leadership Styles and Outcome Patterns for the Nursing Workforce and Work Environment: A Systematic Review.” International Journal of Nursing Studies 85: 19–60. 10.1016/j.ijnurstu.2018.04.016. 29807190

[inr70130-bib-0006] Fajarini, M. , A. Setiawan , C. M. Sung , et al. 2025. “Effects of Advanced Practice Nurses on Health‐Care Costs, Quality of Care, and Patient Well‐Being: A Meta‐Analysis of Randomized Controlled Trials.” International Journal of Nursing Studies 162: 104953. 10.1016/j.ijnurstu.2024.104953. 39586168

[inr70130-bib-0007] Fukuzaki, T. , and N. Iwata . 2024. “The Moderating Role of the Five‐Factor Model of Personality in the Relationship Between Job Demands/Resources and Work Engagement: An Online Cross‐Sectional Study.” Behavioral Sciences (Basel) 14, no. 10: 936. 10.3390/bs14100936. PMC11505521 39457808

[inr70130-bib-0008] García‐Sierra, R. , J. Fernández‐Castro , and F. Martínez‐Zaragoza . 2016. “Work Engagement in Nursing: An Integrative Review.” International Journal of Nursing Studies 53: 312–334. 10.1016/j.ijnurstu.2015.10.010. 26032875

[inr70130-bib-0009] Heinen, M. M. , T. van Achterberg , R. Schwendimann , et al. 2013. “Nurses' Intention to Leave Their Profession: A Cross‐sectional Observational Study in 10 European Countries.” BMC Health Services Research 13: 318. 10.1186/1472-6963-13-318. 23107005

[inr70130-bib-0010] ICN . 2020. Guidelines on Advanced Practice Nursing. International Council of Nurses.

[inr70130-bib-0011] Ida, M. , and H. Fukuda . 2004. “The Effect of Workplace Support for Nurses in Terms of Burnout Response.” Journal of the Faculty of Psychology at Rissho University 1: 77–88.

[inr70130-bib-0012] Inoue, A. , N. Kawakami , T. Shimomitsu , et al. 2014. “Development of a Short Questionnaire to Measure an Extended Set of Job Demands, Job Resources, and Positive Health Outcomes: The New Brief Job Stress Questionnaire.” Industrial Health 52, no. 3: 175–189. 10.2486/indhealth.2013-0185. 24492763 PMC4209588

[inr70130-bib-0013] Japan Nursing Association . 2025. Statistics of Certified Nurses in Japan. https://www.nurse.or.jp/. (in Japanese).

[inr70130-bib-0014] Keyko, K. , G. G. Cummings , O. Yonge , and C. A. Wong . 2016. “Work Engagement in Professional Nursing Practice: A Systematic Review.” International Journal of Nursing Studies 61: 142–164. 10.1016/j.ijnurstu.2016.06.003. 27351831

[inr70130-bib-0015] Kuribayashi, K. , A. Inagaki , K. Imamura , and N. Kawakami . 2025. “Effects of Interventions Aimed at Improving Nurses' Work Engagement in the Workplace: A Systematic Review and Meta‐Analysis Protocol.” BMJ Open 15, no. 1: e085934. 10.1136/bmjopen-2024-085934. PMC11751824 39788773

[inr70130-bib-0016] Lesener, T. , B. Gusy , and C. Wolter . 2019. “The Job Demands–Resources Model: A Meta‐Analytic Review of Longitudinal Studies.” Work Stress 33, no. 1: 76–103. 10.1080/02678373.2018.1529065.

[inr70130-bib-0017] Naef, R. , P. Brysiewicz , N. S. McAndrew , et al. 2021. “Intensive Care Nurse‐Family Engagement From a Global Perspective: A Qualitative Multi‐site Exploration.” Intensive & Critical Care Nursing 66: 103081. 10.1016/j.iccn.2021.103081. 34116886

[inr70130-bib-0018] Rhoades, L. , and R. Eisenberger . 2002. “Perceived Organizational Support: A Review of the Literature.” Journal of Applied Psychology 87, no. 4: 698–714. 10.1037/0021-9010.87.4.698. 12184574

[inr70130-bib-0019] Sato, K. , K. Kubota , and Y. Suenaga . 2023. “Relationship Between Workplace Support Toward Certified Nurses and Work Engagement.” Journal of the Japan Academy of Nursing Administration and Policies 27, no. 1: 150–160. (in Japanese).

[inr70130-bib-0020] Satoh, M. , N. Sato , N. Tamura , and A. Fujimura . 2024. “Psychological Safety in Enhancing the Competence of Nurse Educators Among Early‐Career Nursing Faculty in Japan: A Cross‐Sectional Study.” International Journal of Nursing Studies Advances 7: 100254. 10.1016/j.ijnsa.2024.100254. 39534885 PMC11554630

[inr70130-bib-0021] Schaufeli, W. B. , A. Shimazu , J. Hakanen , M. Salanova , and H. De Witte . 2019. “An Ultra‐short Measure for Work Engagement: The UWES‐3 Validation Across Five Countries.” European Journal of Psychological Assessment 35: 577–591. 10.1027/1015-5759/a000430.

[inr70130-bib-0022] Shimazu, A. , W. B. Schaufeli , S. Kosugi , et al. 2008. “Work Engagement in Japan: Validation of the Japanese Version of the Utrecht Work Engagement Scale.” Applied Psychology: An International Review 57: 510–523. https://iaap‐journals.onlinelibrary.wiley.com/doi/10.1111/j.1464‐0597.2008.00333.x.

[inr70130-bib-0023] Simpson, M. R. 2009. “Engagement at Work: A Review of the Literature.” International Journal of Nursing Studies 46, no. 7: 1012–1024. 10.1016/j.ijnurstu.2008.05.003. 18701104

[inr70130-bib-0024] Slade, M. , S. Rennick‐Egglestone , C. Robinson , et al. 2024. “Effectiveness and Cost‐Effectiveness of Online Recorded Recovery Narratives in Improving Quality of Life for People With Psychosis Experience (NEON Trial): A Pragmatic Randomised Controlled Trial.” The Lancet Regional Health – Europe 47: 101101. 10.1016/j.lanepe.2024.101101. 39507771 PMC11539663

[inr70130-bib-0025] Tsuchihashi, H. , T. Yamaguchi , Y. Yamada , T. Koyama , and Y. Matsunari . 2024. “Factors Associated With Work Engagement of Nurses in the Radiology Department, Japan: A Cross‐Sectional Study.” PeerJ 12: e18426. 10.7717/peerj.18426. 39575172 PMC11580675

[inr70130-bib-0026] Wanning, J. , D. Wan , F. Xueting , G. Liqian , and H. Wenwen . 2024. “Analysis of the Mediating Effect of Social Status Perception on Professional Identity and Work Engagement of Blood Purification Nurses.” Scientific Reports 14, no. 1: 30722. 10.1038/s41598-024-80120-y. 39730464 PMC11681099

[inr70130-bib-0027] Yang, Y. , K. Hatanaka , K. Takahashi , and Y. Shimizu . 2023. “Relationship Among the Nursing Practice Environment, Occupational Career, and Work Engagement of Chinese Nurses Employed in Japan: A Cross‐sectional Study.” International Journal of Nursing Studies Advances 5: 100166. 10.1016/j.ijnsa.2023.100166. 38746593 PMC11080423

